# Chloroplast genomes of four *Carex* species: Long repetitive sequences trigger dramatic changes in chloroplast genome structure

**DOI:** 10.3389/fpls.2023.1100876

**Published:** 2023-01-26

**Authors:** Shenjian Xu, Ke Teng, Hui Zhang, Kang Gao, Juying Wu, Liusheng Duan, Yuesen Yue, Xifeng Fan

**Affiliations:** ^1^ Institute of Grassland, Flowers, and Ecology, Beijing Academy of Agriculture and Forestry Sciences, Beijing, China; ^2^ College of Plants and Technology, Beijing University of Agriculture, Beijing, China

**Keywords:** molecular evolution, plastome, plastid genome, repeats, rearrangement, third-generation sequencing, *Carex*, Cyperaceae

## Abstract

The chloroplast genomes of angiosperms usually have a stable circular quadripartite structure that exhibits high consistency in genome size and gene order. As one of the most diverse genera of angiosperms, *Carex* is of great value for the study of evolutionary relationships and speciation within its genus, but the study of the structure of its chloroplast genome is limited due to its highly expanded and restructured genome with a large number of repeats. In this study, we provided a more detailed account of the chloroplast genomes of *Carex* using a hybrid assembly of second- and third-generation sequencing and examined structural variation within this genus. The study revealed that chloroplast genomes of four *Carex* species are significantly longer than that of most angiosperms and are characterized by high sequence rearrangement rates, low GC content and gene density, and increased repetitive sequences. The location of chloroplast genome structural variation in the species of *Carex* studied is closely related to the positions of long repeat sequences; this genus provides a typical example of chloroplast structural variation and expansion caused by long repeats. Phylogenetic relationships constructed based on the chloroplast protein-coding genes support the latest taxonomic system of *Carex*, while revealing that structural variation in the chloroplast genome of *Carex* may have some phylogenetic significance. Moreover, this study demonstrated a hybrid assembly approach based on long and short reads to analyze complex chloroplast genome assembly and also provided an important reference for the analysis of structural rearrangements of chloroplast genomes in other taxa.

## 1 Introduction

Chloroplasts are unique organelles of green plants and are the site of photosynthesis (Smith and Keeling, 2015). The chloroplast genome has the characteristics of haploid inheritance, relatively small genome, slow mutation rate, and sufficient polymorphism in plants, making it a suitable source of data for the study of evolution and is widely used in studies of phylogeography, population genetics, phylogenetics, molecular evolution, and genome evolution (Xu et al., 2015; Marcussen and Meseguer, 2017; Zhai et al., 2019). In recent years, with the rapid development of high-throughput sequencing technologies, the assembly of the chloroplast genome of conventional species has become convenient and inexpensive, and phylogenetic reconstruction based on the chloroplast genome has led to a much better understanding of the evolutionary relationships of angiosperms (Gitzendanner et al., 2018; Liang et al., 2020; Li et al., 2021).

Due to selection pressure from photosynthesis, the structure of chloroplast genomes of higher plants is primarily conservative, with a circular structure divided into four major regions: large single copy (LSC) and small single copy (SSC) regions separated by two inverted repeats (IRs) of equal length and sequence inversion complementarity (Daniell et al., 2016; Henriquez et al., 2020). The chloroplast genome of land plants is typically 108-165 kb in size and consists of approximately 80 coding protein genes, 4 ribosomal RNAs (rRNAs), and 30 transporter RNAs (tRNAs) (Mower and Vickrey, 2018).

The chloroplast genome is often very stable in angiosperms, but its variation or structural changes can provide some significance for phylogenetic studies (Rokas and Holland, 2000; Qiu et al., 2006; Civáň et al., 2014). In contrast to the expansion and contraction of the IR region (Blazier et al., 2016; Ruhlman and Jansen, 2018), gene loss, and pseudogenization (Ruhlman and Jansen, 2014; Sudianto and Chaw, 2019; Li et al., 2021), complex rearrangements involving multiple events have attracted much attention due to their rarity (Chumley et al., 2006; Knox, 2014). Some of these highly restructured chloroplast genomes result from the absence of IR regions with stable genomic structures (Palmer and Thompson, 1982; Palmer, 1983), such as *Trifolium subterraneum* (Cai et al., 2008). Chloroplast genomes with IR regions and with a high number of rearrangement events have now been found in the gymnosperms and Eudicots branches of angiosperms, such as Geraniaceae (Weng et al., 2014), Campanulaceae (Knox, 2014), Hypericaceae (Claude et al., 2022), and Cupressaceae (Hirao et al., 2008). Some studies have suggested a relationship between this structural rearrangement and the length and number of repetitive sequences (Cai et al., 2008; Guisinger et al., 2011), although the exact reasons remain unclear. The genus *Carex*, one of the few branches of the monocotyledons with a significantly variable chloroplast genome (Wang, 2011), is an important material for studying structural variation in chloroplast genomes.

With about 2000 species, *Carex* L. (Cyperaceae), which belongs to the Poales, is one of the five largest genera of angiosperms (Villaverde et al., 2020; POWO, 2022). Currently, research progress on the chloroplast genome of *Carex* is relatively slow. Only three chloroplast genomes have been formally reported namely: *Carex agglomerata* (Xun et al., 2021), *C. myosuroides* (Chen et al., 2022), and *C. laevissima* (Ren et al., 2022). These results showed that the chloroplast genome size of *Carex* was larger than that of most other angiosperm species (184,157-188,029 bp), and the GC content was relatively low (33.9-34.5%). In the above studies, the chloroplast genome of *Carex* was not investigated in more detail. Moreover, Wang (2021) noted that it was difficult to assemble the chloroplast genome based on short-read data, including raw data from part of the above studies. This is mainly due to a large number of repetitive sequences in the chloroplast genome, resulting in complex structural rearrangements that make it difficult to verify the structure. We propose that assembly of the *Carex* chloroplast genome should be performed in conjunction with long-read data from third-generation sequencing to facilitate validation and exploration of the complex rearrangement events.

Currently, the conventional method for assembling chloroplast genomes is still based on short-read sequencing (second-generation sequencing), which has the advantage of low cost, high data accuracy, and a large number of corresponding assembly methods and programs (Heather and Chain, 2016; Freudenthal et al., 2020). However, these short reads (no more than 350 bp) can be prone to misalignment, making it difficult to obtain information about heterozygous and repetitive regions of the genome (McKenna et al., 2010). In addition, it is difficult to identify structural variations or haplotypic structures using only short reads (Scarcelli et al., 2016). The long reads of third-generation single-molecule sequencing technology provide a solution for complex chloroplast genomes. Pacbio SMART DNA sequencing technology can achieve average read lengths of up to 20 kb and reduce the initial error rate to less than 1% (Rhoads and Au, 2015) and Oxford Nanopore platform can deliver read lengths greater than 20 kb based on high-quality DNA material, with error rates of reads as low as 0.5% by integrating read correction *via* POA graphs into an assembly pipeline and using Nanopolish software (Scheunert et al., 2020). In summary, we have attempted to assemble contigs based on second-generation sequencing data and reconcile complex rearranged regions with third-generation sequencing data to complete the assembly of complex chloroplast genomes such as *Carex*.

The following questions are addressed in this study: (1) Can the challenge of assembling the chloroplast genome of *Carex* be solved by combining second and third-generation sequencing? (2) What are the characteristics of the chloroplast genome of *Carex* and what structural variations occur? (3) What are the possible causes of these variations?

## 2 Materials and methods

### 2.1 Taxon sampling and DNA sequencing

Four taxa representing the four subgenera of *Carex* were selected for this study (**Supplementary Table S1**, Global Carex Group et al., 2021). For *Carex breviculmis*, *C. lithophila* and *C. siderosticta* fresh leaves were collected from the living collection of grasses and sedges of the Beijing Academy of Agriculture and Forestry Sciences. Total genomic DNA was isolated using the CTAB method (Doyle and Doyle, 1987). The extracted total genomic DNA was used for library construction with 2 × 150 bp and 20 kb size libraries and then sequenced on the Illumina Hiseq 4000 platform and Oxford Nanopore PromethION platform for the short and long reads, respectively, at BenaGen (Wuhan, China, www.benagen.com). In addition, *C. littledalei* data are based on the raw data in GenBank (Access number SRR10513805 is second-generation sequencing - Illumina data, Access number SRR9056895 is third-generation sequencing - Pacbio SMRT data).

### 2.2 Chloroplast genome assembly and annotation

We followed previous approaches (Wang et al., 2021) and took advantage of the GetOrganelle pipeline and second- and third- generation sequencing data to achieve high-quality assembly of the complex chloroplast genomes of *Carex*. First, we used GetOrganelle (Jin et al., 2020) (https://github.com/Kinggerm/GetOrganelle) to extract the Illumina sequencing data belonging to the chloroplast and assembled it into a unitig graph that was manually optimized using Bandage software (Wick et al., 2015) to eliminate mitochondria- and nucleus-derived unitig nodes. Subsequently, corrected third-generation sequencing reads (*Carex littledalei* from the PacBio platform; *C. breviculmis*, *C. lithophila* and *C. siderosticta* from the Oxford Nanopore platform) were mapped onto the graph using the minimap2 tool (Li, 2018), and chloroplast-derived long reads were extracted. Then, the repeats on the graph were resolved by alignment with these long reads and circular DNA was constructed. Finally, we checked the assembly results using Geneious Prime (Kearse et al., 2012) to assign the Illumina sequencing reads to the assembled chloroplast genome. The complete chloroplast genome sequences were annotated using CPGAVAS2 (Shi et al., 2019), followed by tRNAscan-SE (Chan et al., 2021) and HS-BLASTN (Chen et al., 2015) for tRNA and rRNA annotation of the chloroplast genome, respectively. Finally, Apollo v2.6.6 (Dunn et al., 2019) was used to correct annotation errors in the chloroplast genomes individually. Illustrations of the four chloroplast genomes were drawn using OGDRAW software (https://chlorobox.mpimp-golm.mpg.de/OGDraw.html).

### 2.3 Codon usage analysis

Codon usage bias was assessed using relative synonymous codon usage (RSCU) correspondence analysis. RSCU scores the 64 vital synonymous codons by calculating the ratio between the actually observed value and the average synonymous codon usage (Wu et al., 2007). The frequency of codon usage is derived by comparing the RSCU value to 1. For example, if the RSCU value is greater than 1, it means a particular codon is used more frequently than other codons (Nabeel-Shah et al., 2014). The protein-coding genes (PCGs) of the chloroplast genome were extracted using Phylosuite software (Zhang et al., 2020). Their protein-coding genes were analyzed for codon preference using Mega v 7.0 (Kumar et al., 2016) and RSCU values were calculated.

### 2.4 Repetitive sequences analysis

Microsatellites were determined using MISA-web (Beier et al., 2017), with a minimum threshold of seven nucleotides for mononucleotide repeats: four for di- and three each for tri-, tetra-, penta-, and hexanucleotide repeats. Dispersal repeats were determined using the REPuter program (Kurtz et al., 2001, minimum repeat size 40 bp) and ROUS Finder (Wynn and Christensen, 2019, default parameters); the program Tandem evaluated Tandem Repeats Finder (TRF) (Benson, 1999) using the default parameters.

### 2.5 Comparative analysis of the chloroplast genome

Since previously published chloroplast genome sequences of *Carex* have not been validated based on three-generation data, and it is difficult to reassemble them based on their raw data, only the four species involved in this study were subjected to comparative genomic analysis. We used mVISTA (Frazer et al., 2004) for the synteny analysis of four *Carex* chloroplast genomes using *C. siderosticta* as a reference, with default parameters and LAGAN and Shuffle-LAGAN mode. Multiple genome alignment was performed using Mauve Alignment of Geneious with default parameters (Darling et al., 2004).

### 2.6 Phylogenetic analysis

To elucidate the phylogeny of *Carex*, 18 chloroplast genome data were downloaded from the NCBI Organelle Genome Resources database (**Supplementary Table S1**) and re-annotated using the Plastid Genome Annotator and manually reviewed in Geneious. Phylogenetic analysis was performed among 22 samples using *Oryza sativa* as the outgroup. Because *Carex* has a large number of structural rearrangements, phylogenetic tree reconstruction was performed using conserved protein-coding genes (PCGs) that were first aligned multiple times using MAFFT software (Katoh and Standley, 2013). Subsequently, these alignments were modified to trim off the gap using Trimal software (Capella-Gutierrez et al., 2009). The ambiguous alignments were removed from the datasets using a Python script (https://github.com/HeJian151004/chloroplast_genome_alignment) (He et al., 2019).

Both the maximum likelihood (ML) method and the Bayesian inference (BI) method were used for phylogenetic reconstruction. The ML tree for each dataset was generated by RAxML v.8.1.17 (Stamatakis, 2014) using the GTR+G model as suggested in the user manual. Bootstrap percentages were calculated after 1,000 replicates. Bayesian inference for each data set was performed using MrBayes v3.2.3 (Ronquist and Huelsenbeck, 2003). The substitution models and data partitions of the complete chloroplast genome dataset for Bayesian analysis were determined using PartitionFinder v2.1.1 (Lanfear et al., 2017). The best scheme was selected according to the Bayesian information criterion (BIC). The partitioning of the other datasets was based on the result of the complete chloroplast genome dataset. For Bayesian inference, the default priorities in MrBayes were used for tree search. Two independent Markov chain Monte Carlo (MCMC) chains were created, each with three heated and one cold chain, for 2,000,000 generations, with tree selection every 100 generations. The first 25% of trees were discarded as burn-in and the remaining trees were used to generate the consensus tree.

In addition, we used Mauve Alignment of Geneious to sequence the entire chloroplast genome described above and then removed the exon sequences from it to subsequently constructed phylogenetic trees based on the intergenic spacer (IGS) sequence using the ML methods.

## 3 Results

### 3.1 Chloroplast genome assembly, organization, and nucleotide composition features

The chloroplast genomes of four *Carex* species were examined by mapping raw data and no gap was found. The genome sizes of the four *Carex* chloroplasts were 213,818 bp (*C. breviculmis*), 208,517 bp (*C. littledalei*), 195,262 bp (*C. siderosticta*), and 181,681 bp (*Carex lithophila*), respectively. The total GC content was 33.4-34.1%. The structure of the *Carex* chloroplast genome is largely consistent with that of other angiosperms, including the LSC region, the SSC region, and two inverted repeat regions (IRa/IRb). The length of the LSC region in the chloroplast genomes of the four *Carex* species assembled in this work was 102,285-103,085 bp with a GC content of 31.8-32.1%; the length of the SSC region was 8,604-8,980 bp with a GC content of 26.9-27.4%; the length of IR was 35,396-51,303 bp with a GC content of 34.2-36.1% (**Table 1**). Thus, the enlargement of the chloroplast genome in *Carex* is mainly due to the enlargement of the IR region, which is 51,303 bp in *C. breviculmis*, resulting in an expanded chloroplast genome of 213,818 bp.

**Table 1:** Detailed information on chloroplast genomes of four *Carex* species.

	*C. breviculmis*	*C. lithophila*	*C. littledalei*	*C. siderosticta*
Total cp genome size (bp)	213,818	181,681	208,517	195,262
Length of inverted repeat region (bp)	51,303	35,396	48,391	41,905
Length of large single copy region (bp)	102,355	102,285	103,085	102,472
Length of small single copy region (bp)	8,857	8,604	8,650	8,980
Total GC content (%)	33.4	34.1	34	34.1
GC content of LSC (%)	32	31.8	32.1	32.1
GC content of IR (%)	34.2	36.1	35.1	35.5
GC content of SSC (%)	27.1	27.4	26.9	27.3
Coding size (bp)	74,058	70,877	70,754	70,810
Noncoding size (bp)	139,760	110,804	137,763	124,452
Total number of genes	102	102	102	102
Number of protein-encoding genes	70	70	70	70
Number of tRNA genes	28	28	28	28
Number of rRNA genes	4	4	4	4
Number of genes duplicated in IR	29	22	22	22

By reviewing the chloroplast genome annotation information using Geneious software, 102 functional genes were encoded in the chloroplast genome of the four *Carex* species studied, including 70 protein-coding genes, 28 tRNA genes, and 4 rRNA genes (**Table 1**). The protein-coding genes included 13 gene families: 11 genes were associated with NADH dehydrogenase subunit coding; 5 genes were associated with photosystem I subunit coding; 15 genes were associated with photosystem II subunit coding; 6 genes were associated with cytochrome b/f complex; 6 genes were associated with ATP synthesis; 7 genes were linked to the large subunit of the ribosome; 10 genes were linked to the small subunit of the ribosome; 3 genes were linked to DNA-dependent RNA polymerase; 4 genes were linked respectively to the formation of the large subunit of Rubisco, maturase, C-type envelope membrane protein, and cytochrome synthesis; 3 genes were of unknown function and open reading. The chloroplast genome of *Carex* species also exhibited partial gene loss, such as *acc*D (involved in acetyl-coenzyme A carboxylase synthesis), *clp*P (encoding a proteolytic subunit of ATP-dependent Clp protease), and *ycf*1 (encoding Tic214 protein). Due to the considerable extension of the IR region, the number of duplicated genes in the IR region of *C. breviculmis* is also significantly higher than in the other three species (29 > 22).

### 3.2 Relative synonymous codon usage

**Figure 1:**
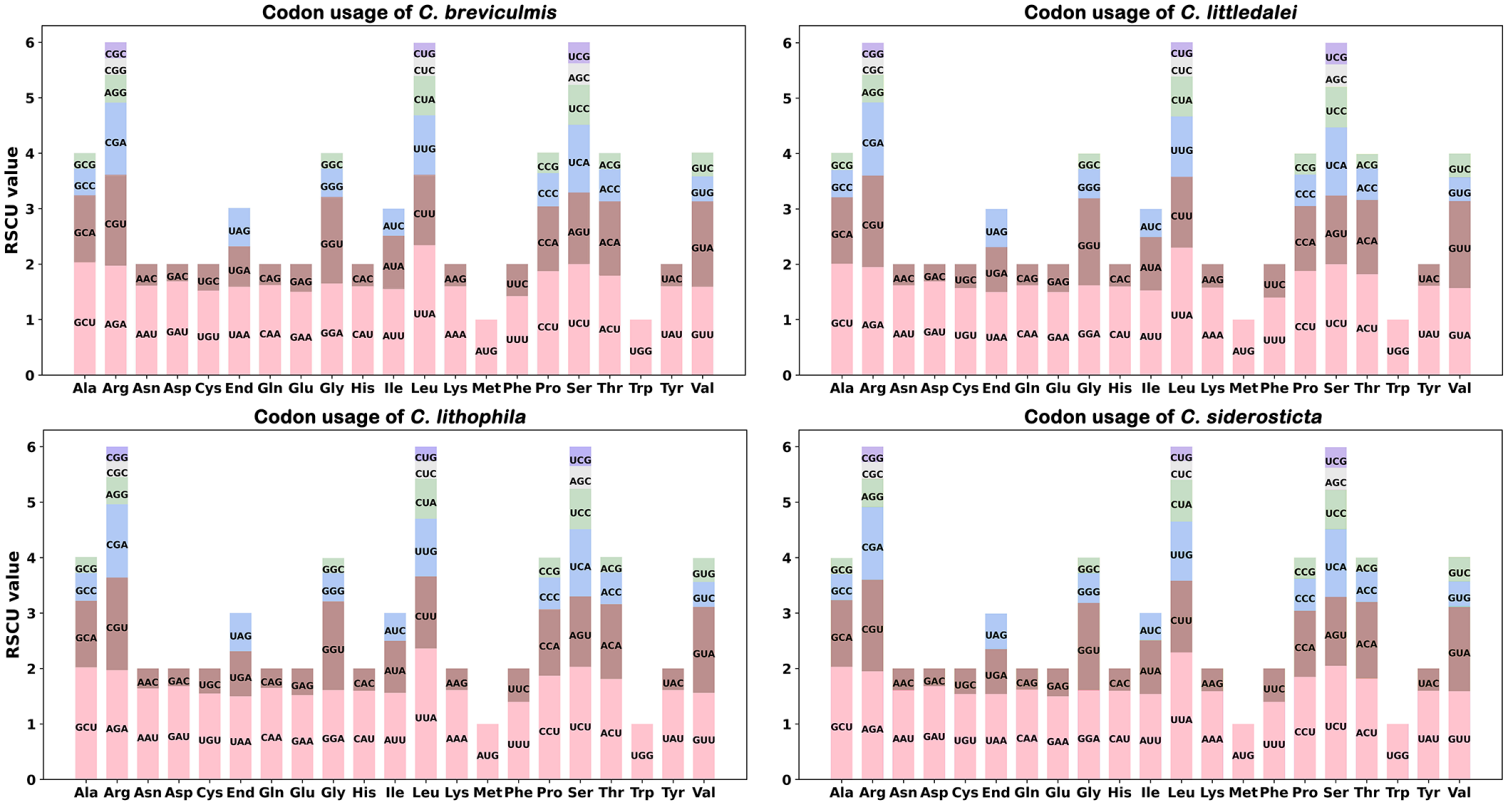
Analysis of codon preferences in the chloroplast genomes of four *Carex* species.

The codon usage frequency of 70 coding genes for 4 *Carex* species was estimated (**Figure 1**). The usage of each amino acid pair is listed in **Supplementary Table S2**. UGA, UAG, and UAA were considered as termination codons, and the RSCU value of UAA was not less than 1.5. For these *Carex* species, we found that UUA-encoded leucine had the highest RSCU value of approximately 2.32 and CUG-encoded leucine had the lowest RSCU value of approximately 0.26.

The chloroplast genomes of all four *Carex* species showed similar codon preferences. For example, leucine (Leu) had a very high preference for UUA with the highest average RSCU value of 2.36 among the chloroplast PCGs, followed by serine (Ser), which also showed a very high preference for UCU codons with an average RSCU value of 2.02. Arginine (Arg), proline (Pro), and threonine (Thr) also had a strong preference for using codons with maximum RSCU values greater than 1.80. In addition, RSCU values of four *Carex* species showed significantly lower nucleotide abundance of G or C than of A or T at the third codon position (14.7 < 45.3); this is similar to other studies of chloroplast genomes (Poczai and Hyvonen, 2017; Ren et al., 2021; Guo et al., 2022).

### 3.3 Long-repeat and simple sequence repeat

**Figure 2:**
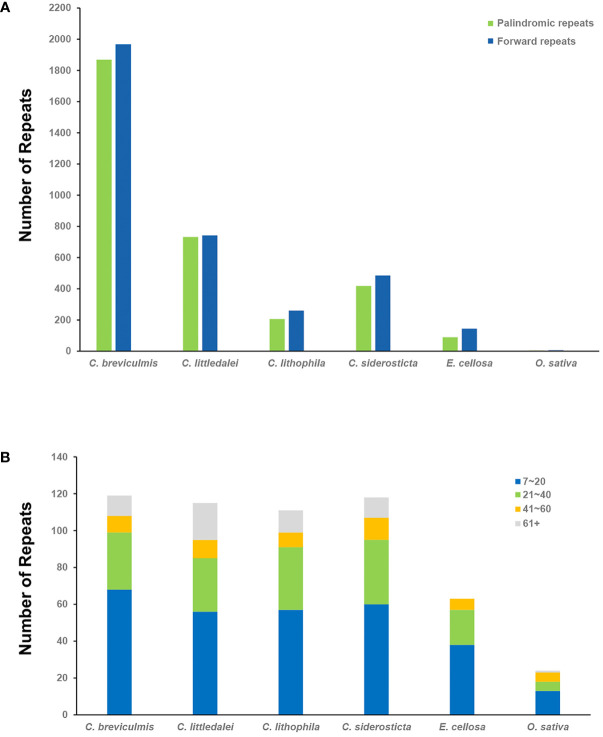
Statistics of repeats in the four chloroplast genomes of *Carex* and its relatives. **(A)** The number of different dispersal repeats in six chloroplast genomes using the REPuter program. P, Palindromic, F, Forward; **(B)** The number of tandem repeat rearrangements in the six chloroplast genomes and the proportion of their length range.

In this study, the dispersal repeats of *Carex* and its close relatives *Eleocharis cellulosa* (Cyperaceae) and *Oryza sativa* (Poaceae) were analyzed using the REPuter program (Kurtz et al., 2001), and two types of repeats were detected in all species: Forward repeats and palindromic repeats (**Figure 2A**, **Supplementary Table S3**). In this study (parameter settings: Hamming distance = 1, minimum repeat = 40 bp), 466-3834 dispersal repeats were detected in four species of *Carex*, 234 in *E. cellulose*, and only 11 in *O. sativa*. The tandem repeats also showed similar results, with 95-108 tandem repeats in the four species of *Carex*, significantly higher than in *E. cellulosa* (63) and *O. sativa* (23). These were mainly observed in 60 bp+ long repeats: the number of tandem repeats over 60 bp in the four species of *Carex* accounted for 10.19-21.05%, significantly higher than their proportions in *E. cellosa* and *O. sativa* (0-4.35%) (**Figure 2B**, **Supplementary Table S5**).

**Figure 3:**
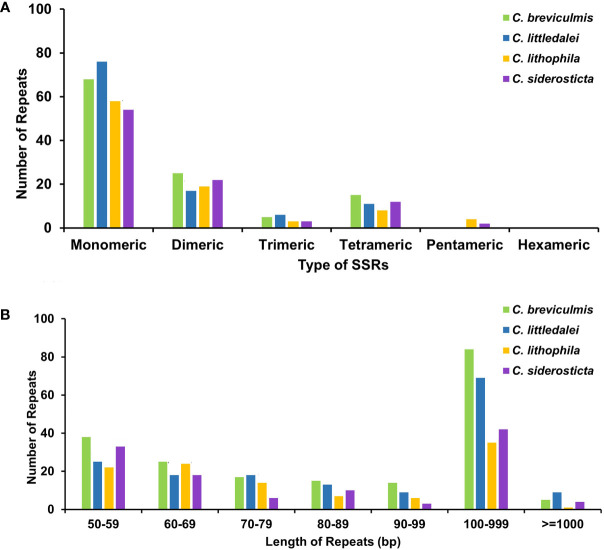
Statistics of the repeats in the four *Carex* chloroplast genomes. **(A)** Analysis of microsatellite repeats. **(B)** Analysis of dispersal repeats based on ROUS Finder.

To better represent the situation of repeat sequences in *Carex*, i.e., excluding redundant repeats with overlapping regions, we used the software ROUS finder to count the dispersal repeats of *Carex* species (Wynn and Christensen, 2019). The four *Carex* species had 198, 178, 114, and 123 sets of repeats longer than or equal to 50 bp, with 89, 78, 36, and 46 repeats longer than 100 bp (**Figure 3B**, **Supplementary Table S4**), respectively, with *Carex breviculmis* having the largest number of repeats longer than 100 bp with a total length of 78,915 bp, representing 36.91% of the total length of the chloroplast genome.

Simple sequence repeats (SSRs), also called microsatellite sequences, are repeat sequences consisting of 1-6 bp linked in tandem as repeat units and are important for the study of plant populations (Powell et al., 1995). SSRs are widely distributed in chloroplast genomes, play a key role in species identification, and are used as important genetic markers to study population genetics and evolution (Zane et al., 2002; Yan et al., 2019). A total of 93-113 SSRs were detected in the chloroplast genomes of four *Carex* species (**Supplementary Table S6**), with the percentage of SSRs in monomeric and dimeric forms ranging from 81.72% (*C. siderosticta*) to 84.55% (*C. littledalei*) (**Figure 3A**). The most common types of dinucleotide repeats were TA (*C. breviculmis*, *C. lithophila*) and AT (*C. littledalei*, *C. siderosticta)*. Hexa-nucleotide repeats were not detected in any of the four *Carex* species, and only pentanucleotide repeats were found in the chloroplast genomes of *C. lithophila* and *C. siderosticta*.

### 3.4 Structural variation in the chloroplast genome of *Carex*

**Figure 4:**
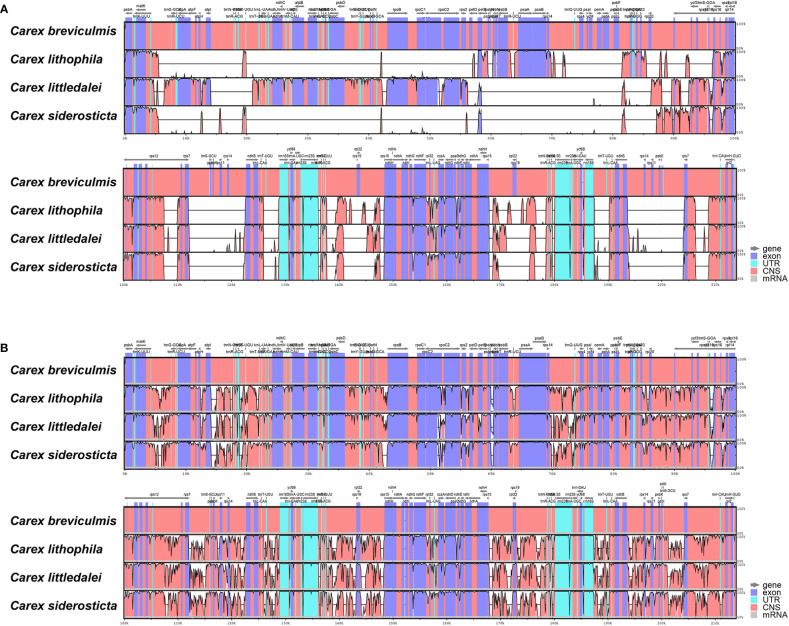
Sequence alignment of four *Carex* species using the program mVISTA. A cut-off value of 70% similarity was used for the plot, and the Y scale indicates the percent similarity between 50 and 100%. Blue represents coding regions and pink represents non-coding regions. **(A)** LAGAN method; **(B)** Shuffle LAGAN method.

The chloroplast genome sequence of *Carex breviculmis* was used as a reference sequence to show analogy between the genomic sequences of four *Carex* chloroplast genomes using mVISTA analysis. The results of the LAGAN-based and shuffle-LAGAN alignment programs are quite different (**Figure 4**), suggesting that there are many structural rearrangements and fragment inversions in *Carex*. Compared to the LSC and IR regions, the SSC and its proximal regions of *Carex* species showed higher consistency in gene order.

**Figure 5:**
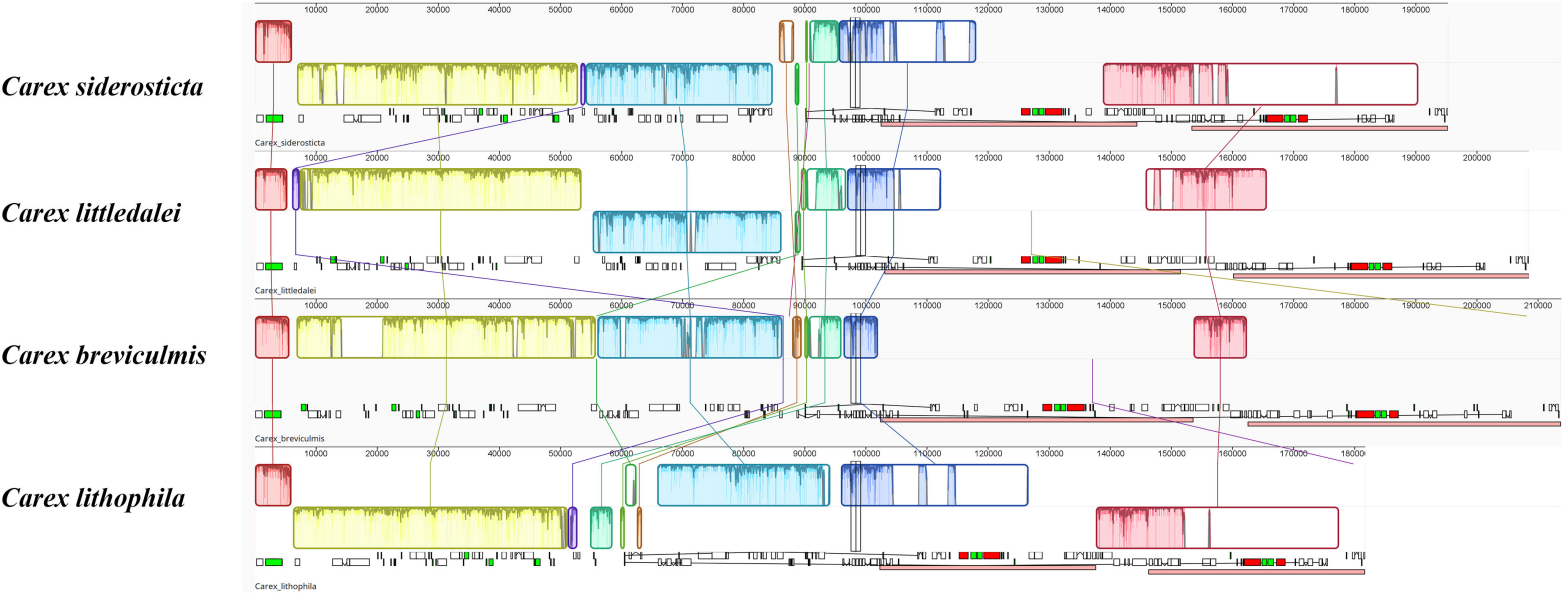
Mauve alignment of four *Carex* chloroplast genomes. Complete chloroplast genome sequences were aligned in Geneious using the Mauve algorithm for linear comparison of rearrangements across the *Carex*. Locally collinear blocks (LCBs) are coloured to indicate syntenic regions. Histograms within each block represent the degree of sequence similarity. Inversions resulting in strand change are represented as offset LCBs (below). The small boxes below each chloroplast genome indicate genes; upper and lower boxes are transcribed counterclockwise and clockwise, respectively. Red boxes indicate ribosomal RNA genes.

**Figure 6:**
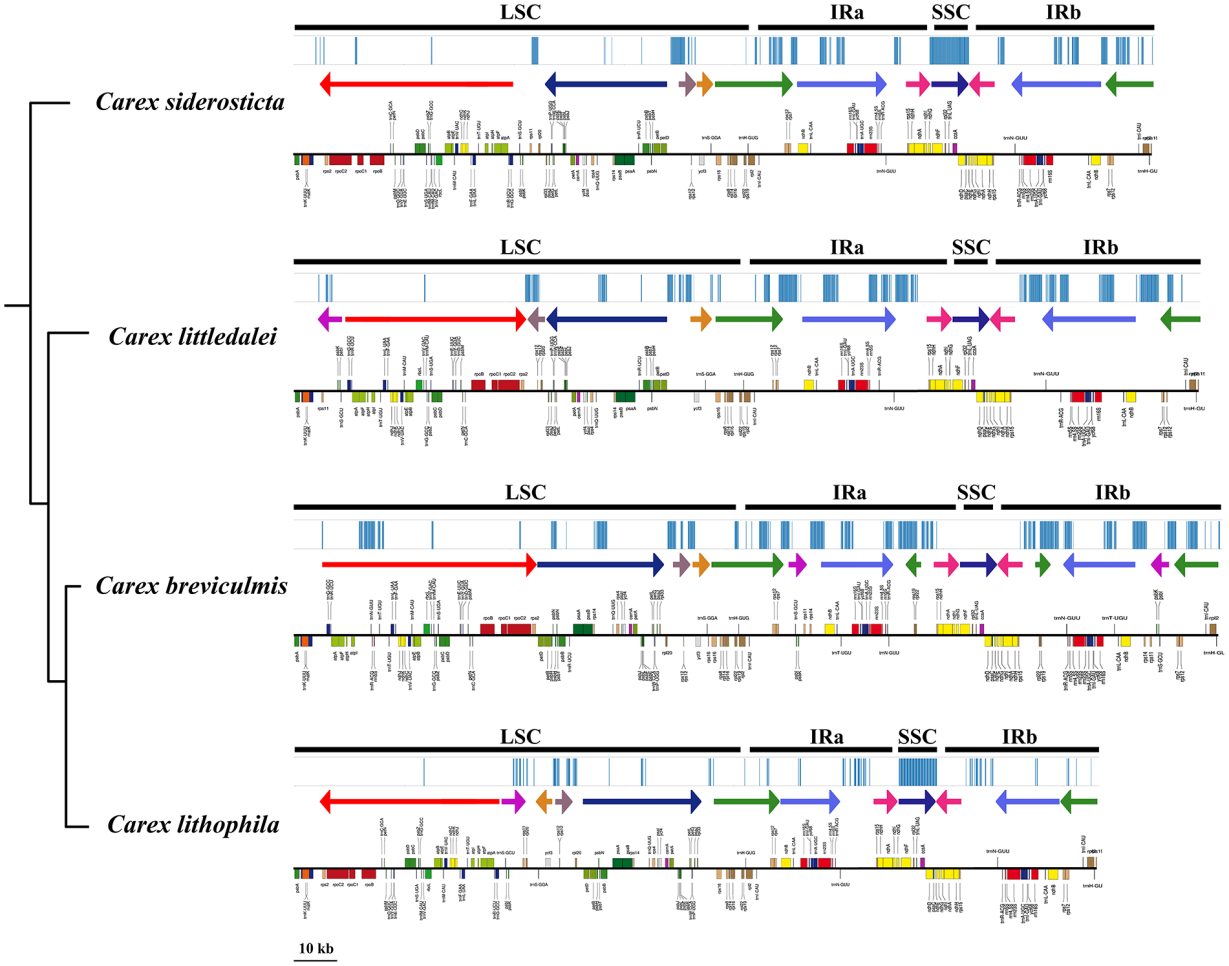
Comparative analysis of differences in chloroplast genomes of four *Carex* species. Bayesian consensus trees based on PCGs are placed on the left. The upper part of the image of each species shows the location of repeats (> 60 bp) in the chloroplast genome; the middle part shows the syntenic regions (arrows) and their directions, arrows of the same color represent syntenic regions between the different species; the lower part shows the map of the chloroplast genome drawn with OGDRAW software.

Alignment of the whole chloroplast genome of four *Carex* species was performed in Mauve Alignment of Geneious. The local collinear blocks (LCBs) identified by Mauve alignment were color-coded to identify genome rearrangements (**Figure 5**). The comparison clearly shows that there are a large number of structural rearrangements in the chloroplast genomes of the four *Carex* species. Structural rearrangements of the chloroplast genome occur not only in *Carex* but also in Cyperaceae and Poales (**Supplement Figure S2**).To better characterize the structural variation of the four *Carex* species, we mapped the syntenic regions (shown as arrows in **Figure 6**) in their chloroplast genomes based on gene order. As **Figure 6** shows, the chloroplast genome structures of the four *Carex* species are very complex with many syntenic regions. Not only do these syntenic regions show altered order between species, but the whole order of genes within the syntenic regions is often altered (the inverted arrow regions indicate that the order of genes is reversed). We marked the positions of the repeats above 60 bp using the Geneious software, shown above the arrows in **Figure 6**. The denser the blue line segment, the more repeats are at that location. It is noteworthy that the endpoints of the rearrangements strongly overlap with the common repetitive sequences of the species, such as *rps*2-*pet*D, *rpl*33-*rps*16, *rps*12-*ndh*B, *rrn*5S-*ndh*H in *C. breviculmis*. Thus, we conclude that the complex chloroplast genome structure of *Carex* species is closely related to the high proportion of repetitive sequences mentioned earlier. This is also an important reason for the difficulties in assembling the *Carex* chloroplast genome based on Illumina data in previous studies, where assembly based on short reads was easily hampered by numerous repeats between syntenic regions.

### 3.5 Phylogenetic relationships in *Carex*

**Figure 7:**
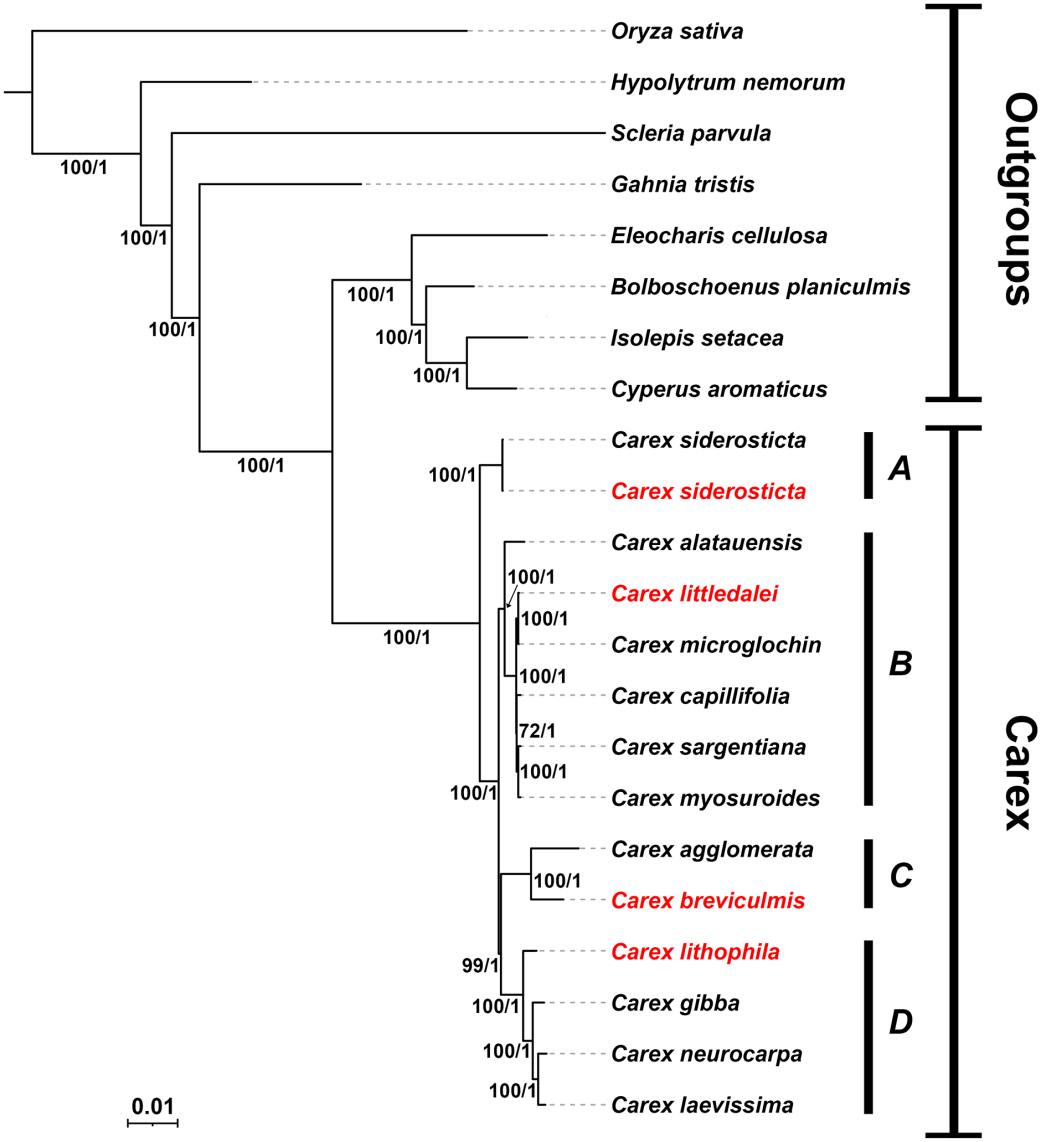
Bayesian consensus tree of *Carex* species based on PCGs. Bootstrap values of maximum likelihood (ML) and predictive probability values (PP) are given at each branches. Chloroplast genomes assembled in this study are highlighted in red. **(A)** C subg. *Siderosticta*; **(B)** C subg. *Euthyceras*; **(C)** C subg. *Carex*; **(D)** C subg. *Vignea*.

Because of the large number of structural rearrangements in the chloroplast genome of *Carex* and the low colinearity of non-coding regions in this species, it is difficult to align and conduct phylogenetic analysis based on whole chloroplast genome data. Phylogenetic analysis was performed using the union of PCGs of 21 species, with *Oryza sativa* of Poaceae and 7 species of Cyperaceae selected as outgroups. Both ML and BI analyses of the complete chloroplast revealed identical topologies with strong support at each node [ML bootstrap (BS) > 95, Bayesian posterior probabilities (PP) = 1] (**Figure 7**). With our limited sampling, the relationships retrieved nonetheless reflect the relationships in the Cyperaceae family as in Larridon et al. (2021), with *Carex* resolved as sister to the clade *Eleocharis* + *Bolboschoenus* + *Isolepis* + *Cyperus* (BS/PP = 100/1). The genus *Carex* is divided into four branches (clade A, clade B, clade C, clade D) corresponding to *C.* subg. *Siderosticta*, *C.* subg. *Euthyceras*, *C.* subg. *Carex*, and *C.* subg. *Vignea*, which is consistent with the most recent taxonomic system of *Carex* (Global Carex Group et al., 2021). *C. siderosticta* is at the base of *Carex* and taxa formerly belonging to *Kobresia*, such as *C. littledalei*, *C. myosuroides*, are in clade B, which is sister related to clade C + clade D. In this study, phylogenetic relationships in *Carex* based on chloroplast PCGs support the most recent *Carex* taxonomic system and are also consistent with previous phylogenetic relationships constructed based on low copy nuclear orthologous nuclear loci derived from the Cyperaceae-specific HybSeq bait kit. (Villaverde et al., 2020; Global Carex Group et al., 2021). The phylogenetic tree constructed based on the IGS sequences has a very consistent tree-like structure, further supporting the above results (**Supplement Figure S3**).

## 4 Discussion

Although *Carex* is one of the largest genera of angiosperms, there has been a lack of in-depth studies on its chloroplast genome evolution due to its large number of repetitive sequences and relatively complex structural variation. In this study, clear pictures of the chloroplast genome structure of four *Carex* species were obtained by combining second- and third-generation sequencing data (**Supplementary Figure S1**). Although no structural heteroplasmy similar to that of the *Eleocharis* plastome was found in *Carex*, the extreme abundance of repetitive sequences and the complex rearrangements of the chloroplast within this genus provide a valuable model for the study of chloroplast genome variation.

### 4.1 Third-generation sequencing facilitates the assembly of complex chloroplast genomes

For most species, second-generation sequencing has become the primary data source for chloroplast genome assembly due to its easy sequence acquisition, high accuracy, and favorable price. Chloroplast genomes assembled based on Illumina sequencing are regarded as the “gold standard” for data quality and integrity (Scheunert et al., 2020). Freudenthal et al. (2020) analyzed conventional chloroplast assembly methods and concluded that GetOrganelle (Jin et al., 2020) and Fast-Plast (McKain and Wilson, 2017) can provide convenient and rapid assembly methods with accurate Illumina data. However, taxa such as *Pelargonium* (Chumley et al., 2006), *Trifolium* (Sveinsson and Cronk, 2014), and *Carex* are difficult to assemble by conventional assembly methods based on Illumina data due to the presence of abundant repeats and rearrangements. In this case, assembly between contigs of complex chloroplast genomes assembled based on second-generation data in *Carex* is often complex because of the large number of repetitive sequences that are difficult to bridge at the nodes where long repeats occur. Third-generation sequencing technologies generally have longer read lengths that can effectively determine the assembly mode of the above nodes at high coverage and greatly improve the accuracy of complex chloroplast assemblies (Wu et al., 2014; Scheunert et al., 2020).

**Figure 8:**
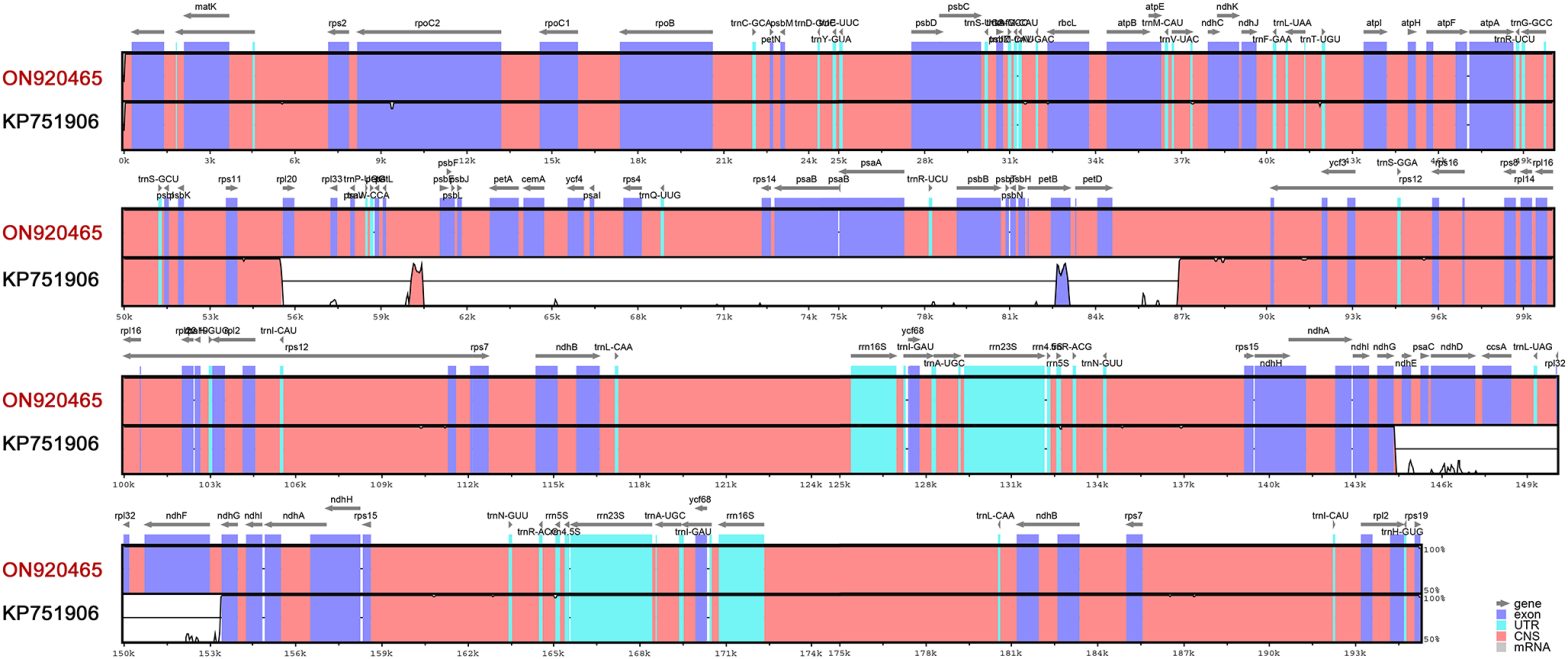
mVISTA identity diagram based on LAGAN alignment for *Carex siderosticta* assembled by two different methods and data sources. ON920465 was used as the reference sequence, which was derived from a hybrid assembly of second and third generation data. A cut-off value of 70% similarity was used for the plot, and the Y scale indicates the percent similarity between 50 and 100%. Blue represents coding regions and pink represents non-coding regions.

**Figure 8** shows the variability of assemblies of the same species (*Carex siderosticta*) based on different datasets and methods. We used the sequence (ON920465) assembled with a hybrid of second- and third- generation data and were able to show higher accuracy in mapping reads as a reference, while the sequence (KP751906) showed some problems with assembly results between *rpl*20-*pet*D, *ndh*D-*ndh*E, and so on. Such discrepancies are probably due to incorrect assembly caused by a large number of repeats. With the rapid development of third-generation sequencing technologies, especially the rapid proliferation of MinION devices that are inexpensive and easy to install in most laboratories (Freudenthal et al., 2020), it is possible to optimize previous assembly results based on Illumina sequences in combination with long read-length sequences to achieve assembly of complex chloroplast genomes and perform in-depth studies on their structural variation features. This study demonstrates a suitable case for the assembly of complex chloroplast genomes with a large number of repetitive sequences that may be helpful for the subsequent assembly of related species.

### 4.2 The chloroplast genome characters of four *Carex* species

All four *Carex* species studied have chloroplast genomes of well above average length (151 kb, Ruhlman and Jansen, 2014), with *C. breviculmis* (213,818 bp) having one of the longest chloroplast genomes of land plants reported to date. In addition to the expansion of the region IR, which occurs in most angiosperms and results in their larger chloroplast genomes, such as in *Pelargonium × hortorum*, *Musa acuminata*, *Cyphia crenata*, etc. (Martin et al., 2013; Weng et al., 2017; Li et al., 2020), the increase in chloroplast genome size in *Carex* is also accompanied by an increase in the number and size of repetitive sequences. Similar results were found in *Eleocharis*, another genus of Cyperaceae (Lee et al., 2020). The gene numbers and species of the four *Carex* species are highly consistent (**Table 1**), and the differences in chloroplast genome length within their genera are mainly due to IR contraction and expansion, with *C. breviculmis* having a significantly expanded IR region (51,303 bp). In conjunction with phylogenetic studies (**Figure 6**), it is likely that chloroplast genomes of *Carex* species undergo multiple expansions and contractions.

The chloroplast GC content of four *Carex* species was relatively low, similar to other taxa in the Cyperaceae (Ren et al., 2021) and significantly lower than in the Poaceae (**Supplementary Table S1**). It is worth noting that IR has a higher GC content than the SC region (**Table 1**) and the IR of the *Carex* species chloroplast genome is significantly expanded (35,396-51,303 bp), so the expansion of IR should have resulted in a higher GC content. The still significantly lower than average overall GC content of *Carex* species is largely due to the widespread repetitive sequences in the intergenic spacer regions within their chloroplast genomes and their extremely low GC content (24.40-26.08%), and such a pattern is also found in the chloroplast genomes of *Eleocharis* (**Supplementary Table S1**, Lee et al., 2020).

In the chloroplast genome, gene loss is a relatively rare genome-shaping event that can provide important information for our understanding of the phylogenetic relationships between genes and between species (Harris et al., 2013). In the present study, we found some events of gene deletion in all four *Carex* species, such as the *acc*D, c*lp*P, and *ycf*1 genes. Previously, the *acc*D gene was thought to be the subject of widespread deletion or pseudogenization in Poales (Harris et al., 2013; Wysocki et al., 2016). However, the evolutionary history of this gene within Cyperaceae remains controversial due to limited and conflicting data (Poczai and Hyvönen, 2017; Lee et al., 2020). None of the four *Carex* species involved in this study contained the *acc*D gene, which is present only in basal taxa within the Cyperaceae (**Supplementary Table S7**), indicating that the *acc*D gene has also undergone at least one gene loss event within the Cyperaceae. A generally accepted explanation for the widespread occurrence of deletions is that *acc*D is located in regions with high mutation rates (Ogihara et al., 1991; Maier et al., 1995). Similarly, gene loss also occurs in other specific coding genes, which often have more SNPs than any other coding motifs, such as *clp*P, *ycf*1, in *Carex*. Such divergent results may be due to aberrant DNA duplication, repair, or recombination during the evolution of their common ancestor (Guisinger et al., 2008; Dugas et al., 2015).

It is now widely accepted that the use of synonymous codons is not random and that analysis of codon preferences can provide valuable information for understanding species adaptation and molecular evolution (Plotkin and Kudla, 2011; Leffler et al., 2012). The chloroplast genomes of four *Carex* species contain 30 high-frequency codons (RSCU > 1) and prefers codons ending in A/T, similar to other closely related taxa (Poczai and Hyvonen, 2017; Chakraborty et al., 2020; Ren et al., 2021; Guo et al., 2022). Variation in codon bias among *Carex* species was not significant, with only minor differences in optimal codons for valine (Val) (GUA in *C. littledalei*, with GUU in *C. breviculmis*, *C. lithophila*, *C. siderosticta*).

### 4.3 Structural variation in the chloroplast genome of *Carex*

It appears that *Carex* species have one of the most restructured chloroplast genomes of angiosperms sequenced to date, with a large number of rearrangement events in their chloroplast genome and corresponding changes in the position and sequence of many genes. The structure of the chloroplast genome of *Carex* is closer to that of taxa such as *Geranium* than to that of *Trifolium*, where the absence of a region IR leads to a large number of rearrangements (Cai et al., 2008). The chloroplast genomes of all four *Carex* species have been shown to have a circular quadripartite structure and also to have a significantly higher number of repetitive sequences than those of closely related taxa, with the differences being greatest for the long repeats (> 60 bp). Furthermore, this study supports the idea that rearrangements in the chloroplast genome are significantly correlated with repeats, especially long repeats, by graphically showing that long repeats occur very frequently at rearrangement endpoints in the chloroplast genome (Weng et al., 2014) (**Figure 6**). Notably, such association is also found in the highly rearrangement genomes of *Pelargonium* (Chumley et al., 2006), *Trifolium* (Sveinsson and Cronk, 2014) and *Trachelium* (Haberle et al., 2008). Our results also show that the number of repeats positively correlates with the degree of rearrangement of the chloroplast genome in *Carex*, with the chloroplast genomes of *C. breviculmis* having the most repeats and also suffering the most extensive rearrangements. Although we are unable to determine the exact mechanism of genome rearrangement, it is reasonable to assume that repeats play an important role in genome rearrangement and sequence divergence through illegitimate recombination and slipped-strand mispairing (Rogalski et al., 2006; Timme et al., 2007; Gray et al., 2009).

Molecular rearrangements in the chloroplast are important because their fixation in the genome during evolution is rare (Downie and Palmer, 1992; Lee et al., 2007). The rearrangement features of the chloroplast genome may also provide some phylogenetic significance (Cosner et al., 2004). This study demonstrated that *Carex siderosticta* and *C. littledalei* are more closely related and show higher chloroplast genome collinearity, with only one reversal in the red arrow region (*atp*A-*rps*2) and one change in position in the brown arrow (*rps*12-*rpl*20) (**Figure 6**). *C. breviculmis* and *C. lithophila* have the reverse order of the other two species in the blue arrow region (*pet*D-*rpl*33) and may have undergone a flip-flop event in their common ancestor. *C. breviculmis* shows the highest level of chloroplast genome rearrangement than in other taxa due to the presence of most repeats, and several additional insertions of collinear regions within the IR region resulting in a significant increase in sequence length. However, the phylogenetic relationships of the four *Carex* species provide only limited information on the evolutionary history of structural variation in their chloroplast genomes, and a more in-depth analysis of the evolution of chloroplast genomes within the genus requires the sequence and structural characterization of additional species.

## 5 Conclusion

In this study, the chloroplast genomes of four *Carex* species were assembled and annotated using Illumina and third generation sequencing (PacBio SMRT and Nanopore) data to provide new insights into the evolution of chloroplast genomes in *Carex*. Compared to conventional species, *Carex* chloroplast genomes are characterized by a large number of repetitive sequences and low GC content. We found that a high frequency of long repeats is found at the rearrangement termini, strongly suggesting that long repeats can induce structural variation in the chloroplast genome.

## Data availability statement

The original contributions presented in the study are publicly available. This data can be found here: GenBank ON920463, ON920464, ON920465, and OP764679. The raw sequencing data were deposited into the NCBI Short Read Archive with the accession number PRJNA901371.

## Author contributions

SX: Conceptualization, methodology, validation, formal analysis, investigation, data curation, writing – original draft, writing – review and editing, visualization, project administration. KT: Methodology, software, validation, and formal analysis. HZ: Investigation, resources, and writing – review and editing. KG: Formal analysis, investigation, and data curation. JW: Formal analysis, and writing – review and editing. LD: Writing – review and editing. YY: Methodology, project administration, and funding acquisition. XF: Writing – review and editing, supervision, project administration, and funding acquisition. All authors contributed to the article and approved the submitted version.

## Supplementary material

Supplementary Figure 1 Chloroplast genome maps of four *Carex* species assembled in this study. The thick lines on the outer circle indicate the inverted repeat regions (IRa and IRb). The innermost trace indicates GC content. Genes on the outside of the map are transcribed clockwise, and genes on the inside of the map are transcribed counterclockwise.

Supplementary Figure 2 Mauve alignment of chloroplast genomes of 8 species in Cyperaceae and *Oryza sativa* (Poaceae). Complete chloroplast genome sequences were aligned in Geneious using the Mauve algorithm for linear comparison of rearrangements across the Cyperaceae. Locally collinear blocks (LCBs) are coloured to indicate syntenic regions. Histograms within each block represent the degree of sequence similarity. Inversions resulting in strand change are represented as offset LCBs (below). The small boxes below each chloroplast genome indicate genes; upper and lower boxes are transcribed counterclockwise and clockwise, respectively. Red boxes indicate ribosomal RNA genes.

Supplementary Figure 3 Maximum likelihood (ML) tree of *Carex* species based on IGS sequence. Bootstrap values of ML are given at each branch. Chloroplast genomes assembled in this study are highlighted in red. A: *C.* subg. *Siderosticta*; B: *C.* subg. *Euthyceras*; C: *C.* subg. *Carex*; D: *C.* subg. *Vignea*.

Supplementary Table 1 Sequence information involved in this study.

Supplementary Table 2 Codon usage bias by chloroplast gene in four *Carex* species.

Supplementary Table 3 Dispersal repeats analysis of the chloroplast genomes of four *Carex* species and two of their relatives using REPuter software.

Supplementary Table 4 Dispersal repeats analysis of the chloroplast genomes of four *Carex* species using ROUS software.

Supplementary Table 5 Tandem repeat sequences in the chloroplast genomes of four *Carex* species and two of their relatives.

Supplementary Table 6 Simple Sequence Repeats (SSRs) in the chloroplast genome of four *Carex* species.

Supplementary Table 7 Statistics of partial gene losses (*acc*D, *clp*P, etc.) in the chloroplast genomes of all species involved in this study.
