# Supplementary figures and images for: Chloroplast genomes of four Carex species: Long repetitive sequences trigger dramatic changes in chloroplast genome structure
Source: Front Plant Sci. 2023 Jan 26;14:1100876. doi: 10.3389/fpls.2023.1100876 (PMC9911286; doi:10.3389/fpls.2023.1100876)

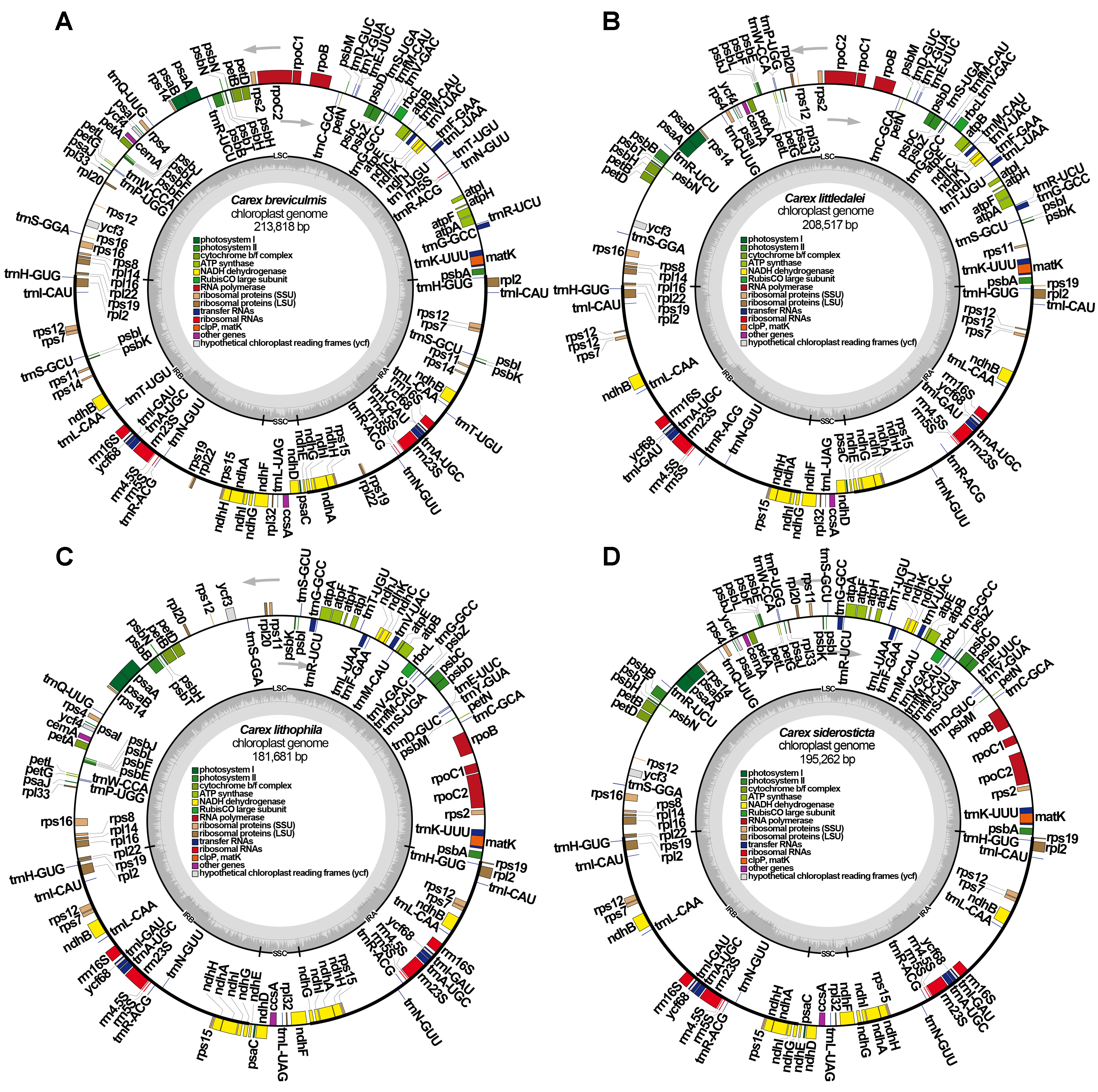

Supplement: Supplementary Figure 1 — Chloroplast genome maps of four Carex species assembled in this study. The thick lines on the outer circle indicate the inverted repeat regions (IRa and IRb). The innermost trace indicates GC content. Genes on the outside of the map are transcribed clockwise, and genes on the inside of the map are transcribed counterclockwise. [file Image_1.jpeg]

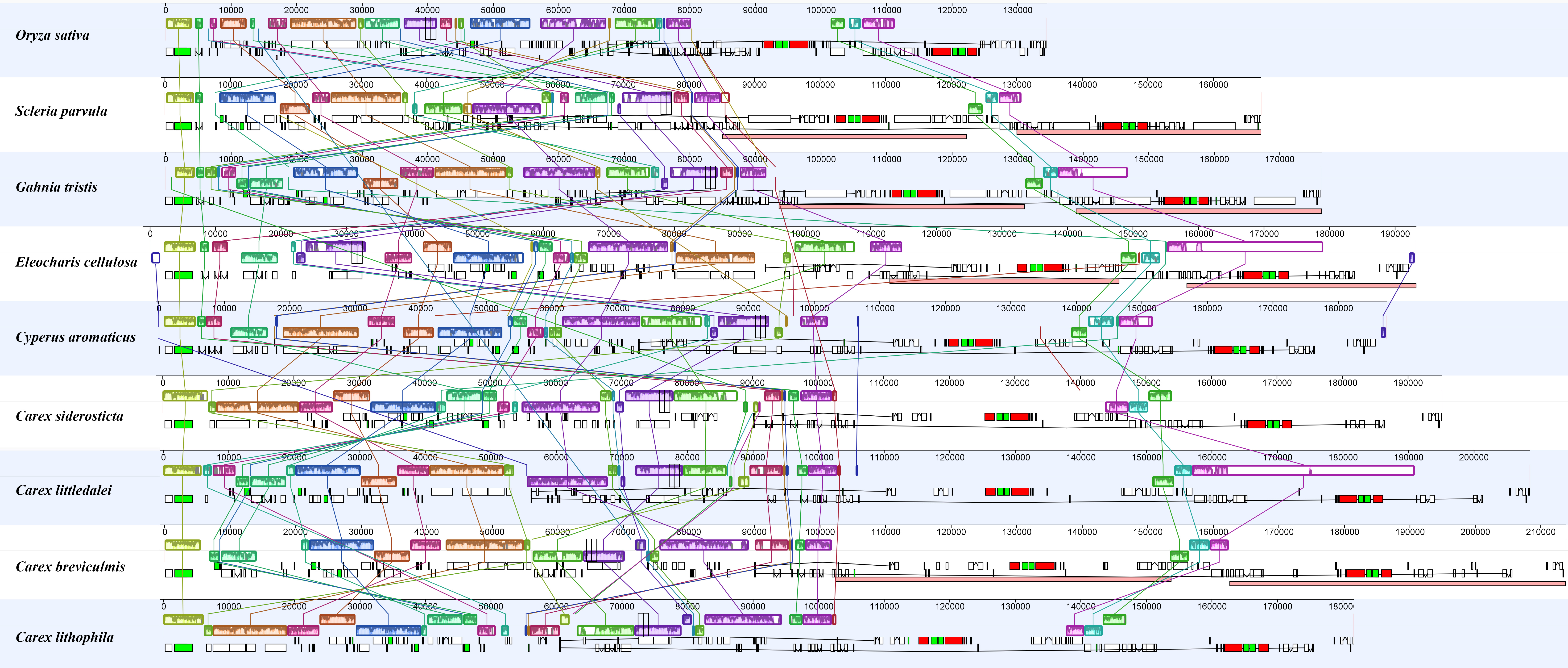

Supplement: Supplementary Figure 2 — Mauve alignment of chloroplast genomes of 8 species in Cyperaceae and Oryza sativa (Poaceae). Complete chloroplast genome sequences were aligned in Geneious using the Mauve algorithm for linear comparison of rearrangements across the Cyperaceae. Locally collinear blocks (LCBs) are coloured to indicate syntenic regions. Histograms within each block represent the degree of sequence similarity. Inversions resulting in strand change are represented as offset LCBs (below). The small boxes below each chloroplast genome indicate genes; upper and lower boxes are transcribed counterclockwise and clockwise, respectively. Red boxes indicate ribosomal RNA genes. [file Image_2.jpeg]

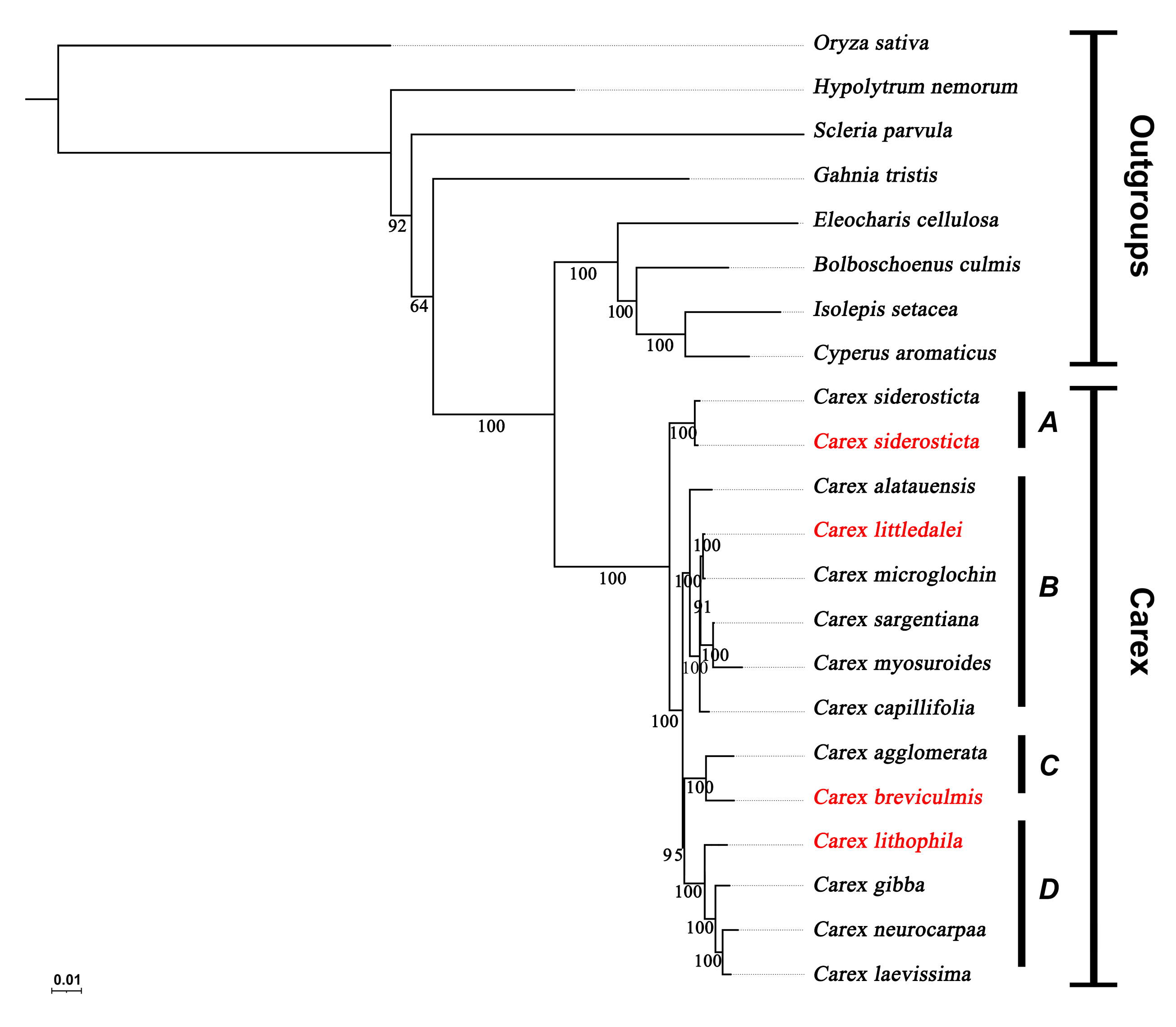

Supplement: Supplementary Figure 3 — Maximum likelihood (ML) tree of Carex species based on IGS sequence. Bootstrap values of ML are given at each branch. Chloroplast genomes assembled in this study are highlighted in red. A: C. subg. Siderosticta; B: C. subg. Euthyceras; C: C. subg. Carex; D: C. subg. Vignea. [file Image_3.jpeg]
